# Specific signature biomarkers highlight the potential mechanisms of circulating neutrophils in aneurysmal subarachnoid hemorrhage

**DOI:** 10.3389/fphar.2022.1022564

**Published:** 2022-11-10

**Authors:** Weipin Weng, Fan Cheng, Jie Zhang

**Affiliations:** Department of Neurology, The Second Xiangya Hospital, Central South University, Changsha, Hunan, China

**Keywords:** transcription profiles, neutrophil, peripheral inflammatory response, aneurymal subarachnoid hemorrhage, machine learning

## Abstract

**Background:** Aneurysmal subarachnoid hemorrhage (aSAH) is a devastating hemorrhagic stroke with high disability and mortality. Neuroinflammation and the immunological response after aSAH are complex pathophysiological processes that have not yet been fully elucidated. Therefore, attention should be paid to exploring the inflammation-related genes involved in the systemic response to the rupture of intracranial aneurysms.

**Methods:** The datasets of gene transcriptomes were downloaded from the Gene Expression Omnibus database. We constructed a gene co-expression network to identify cluster genes associated with aSAH and screened out differentially expressed genes (DEGs). The common gene was subsequently applied to identify hub genes by protein-protein interaction analysis and screen signature genes by machine learning algorithms. CMap analysis was implemented to identify potential small-molecule compounds. Meanwhile, Cibersort and ssGSEA were used to evaluate the immune cell composition, and GSEA reveals signal biological pathways.

**Results:** We identified 602 DEGs from the GSE36791. The neutrophil-related module associated with aSAH was screened by weighted gene co-expression network analysis (WGCNA) and functional enrichment analysis. Several small molecular compounds were predicted based on neutrophil-related genes. MAPK14, ITGAM, TLR4, and FCGR1A have been identified as crucial genes involved in the peripheral immune activation related to neutrophils. Six significant genes (CST7, HSP90AB1, PADI4, PLBD1, RAB32, and SLAMF6) were identified as signature biomarkers by performing the LASSO analysis and SVM algorithms. The constructed machine learning model appears to be robust by receiver-operating characteristic curve analysis. The immune feature analysis demonstrated that neutrophils were upregulated post-aSAH and PADI4 was positively correlated with neutrophils. The NETs pathway was significantly upregulated in aSAH.

**Conclusion:** We identified core regulatory genes influencing the transcription profiles of circulating neutrophils after the rupture of intracranial aneurysms using bioinformatics analysis and machine learning algorithms. This study provides new insight into the mechanism of peripheral immune response and inflammation after aSAH.

## Introduction

Subarachnoid hemorrhage (SAH) is a devastating hemorrhagic cerebrovascular disease with rapid progression, extremely high morbidities, and mortalities, which affects six to nine individuals per 100,000 annually, worldwide (Neifert et al., 2021). Aneurysmal subarachnoid hemorrhage (aSAH) is a leading cause of SAH, accounting for about 85% of all cases (Macdonald and Schweizer, 2017). Despite advances in aneurysm repair as well as improved neurocritical care of SAH patients following aneurysm repair, subarachnoid hemorrhage still has complex and serious complications in survived patients (Lovelock et al., 2010). Although it is an atypical subtype of stroke, aSAH occurs in relatively young patients (mean age 55 years) than ischemic stroke, which imposes considerable social and economic burdens (Jaja et al., 2018). The aSAH therapeutic strategies to date have mainly focused on treating ruptured aneurysms, despite the fact that multiple mechanisms and factors impact clinical outcome after aSAH.

Mounting evidence indicates that systemic immune and inflammatory reactions may play crucial roles in contributing to brain injury and outcome after aSAH (Lucke-Wold et al., 2016; Chaudhry et al., 2018a). Molecules from extravasated blood in the subarachnoid space as damage-associated molecular patterns (DAMPs) trigger an inflammatory cascade and activate the innate immune cells in the central nervous system (Chaudhry et al., 2018b). Subsequently, recruited immune cells in the peripheral circulation mediated by the chemokines release pro-inflammatory cytokines which result in blood-brain barrier (BBB) disruption, injury of oxidative stress, microthrombosis, loss of neurons, and permanent neurological impairment (Schneider et al., 2012; Tso and Macdonald, 2014; McBride et al., 2017; Liu et al., 2018; Schneider et al., 2018; Fumoto et al., 2019; Li et al., 2020; Wu et al., 2021). Multiple studies have provided evidence that increases in pro-inflammatory cytokines have been linked to worse outcomes for patients after aSAH (Lenski et al., 2017; Ridwan et al., 2021). However, the mechanism by which peripheral immune cells participate in systemic inflammation and secondary brain injury remains unclear.

Neutrophils are the most abundant type of leukocyte in human circulation and are crucial peripheral inflammatory cells that rapidly infiltrate the central nervous system during the hemorrhagic event (Friedrich et al., 2011). Previous studies showed that elevated neutrophil was associated with worse mortality and functional outcome in patients with aSAH, as well as mediated cerebral vasospasm and microvascular injury (Chou et al., 2011; Provencio et al., 2011; Zhang et al., 2021). Additionally, neutrophil infiltration in the brain contributes to microglia-mediated cerebral spreading inflammation (Atangana et al., 2017). Instead, neutrophil depletion reduces tissue inflammation and cerebral vasoconstriction of the middle cerebral artery, improving survival in animal models (Friedrich et al., 2011; Provencio et al., 2016; Gris et al., 2019). Nevertheless, the underlying neutrophil effector mechanism requires further elucidation.

**FIGURE 1:**
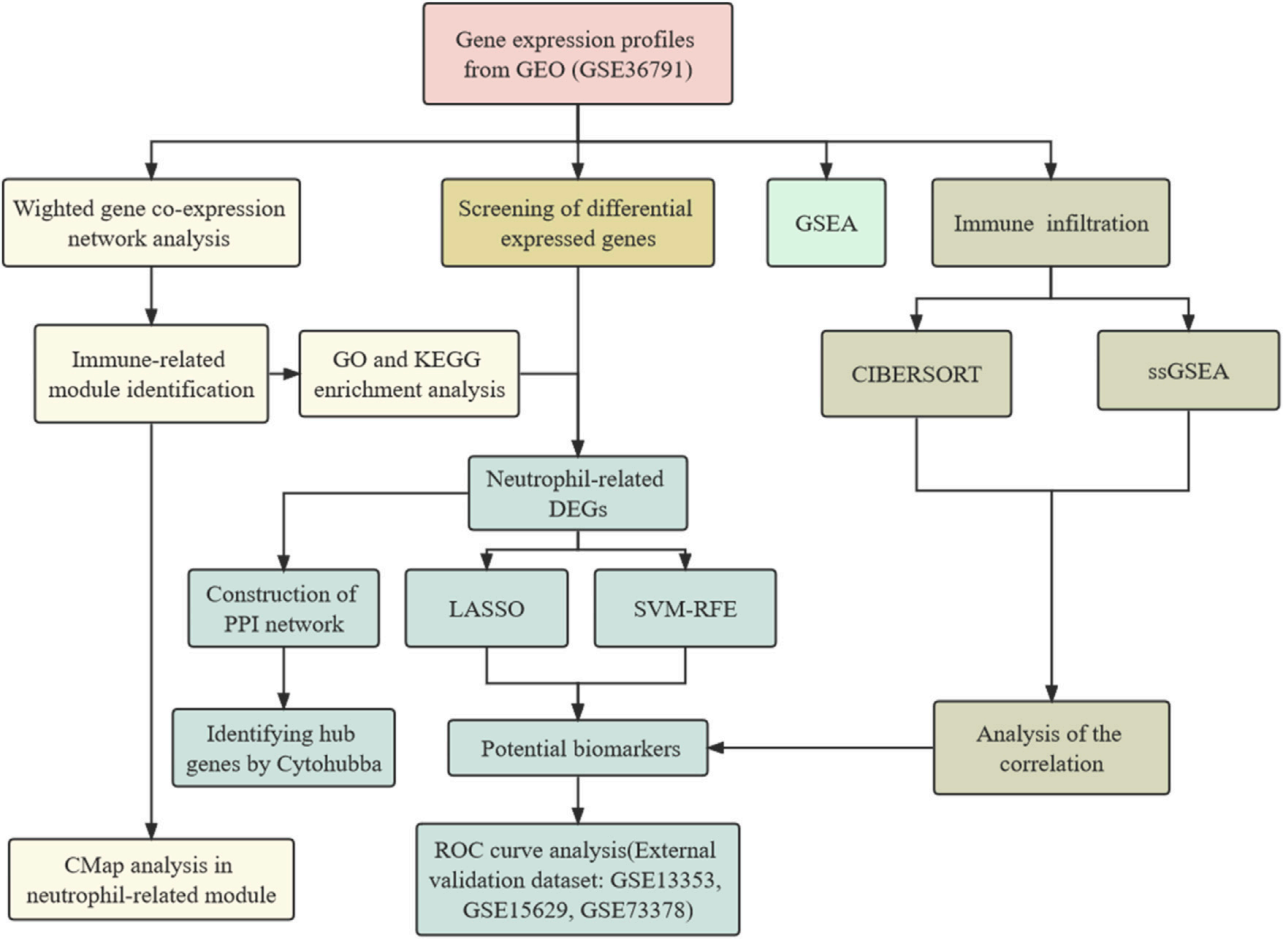
Study flow chart.

Here, we investigated the potential biomarkers and molecular mechanisms in the peripheral blood transcriptome of aSAH patients through bioinformatics data analysis. The process of data preparation and analysis is illustrated as a flow chart in Figure 1. We depicted how the immune cell landscape is perturbed in aSAH. Neutrophil-related module was identified by utilizing the co-expression network. Based on the six signatures screening out using the machine-learning strategies, a neutrophil-related model for aSAH patients was developed and validated its significant diagnostic values for aSAH. Finally, the candidate target drugs therapeutically interfering with neutrophils were identified by the connectivity map (CMap). In summary, the gene expression level analysis could highlight the potential pathways and effector mechanisms linked to the peripheral immune activation of neutrophils, and provide new insights into the development of immunoregulatory treatment for aSAH.

## Materials and methods

### Microarray datasets collection and processing

The gene expression profiles were obtained from Gene Expression Omnibus (GEO) database (http://www.ncbi.nlm.nih.gov/geo/) which is public availability. We downloaded the GSE36791 annotated by GPL10558 (Illumina HumanHT-12 V4.0 expression beadchip) as a Series Matrix File which is the transcriptome in peripheral blood cells of 43 patients with SAH from ruptured aneurysm compared with that of 18 control patients suffering from headaches without intracranial aneurysm (Pera et al., 2013). Meanwhile, the gene expression profiles of aSAH GSE73378, GSE15629, and GSE13353 were obtained as external validation datasets. R software (version 4.1.1) was used to perform the bioinformatics analysis. The probe IDs in each dataset were then converted into gene symbols based on their platform annotation. The expression dates were normalized and log2 transformed.

### Differential expression gene identification

The limma algorithm was adopted to identify differentially Expressed Genes (DEGs) between 43 aSAH and 18 control samples (Ritchie et al., 2015). The genes that meet the screening criteria [|log2 FC (fold change) | > 0.5, false discovery rate (FDR) adjusted *p*-value< 0.05] were considered as the cut-off for the DEGs. Then, we drew the cluster heat map and volcano plot to visualize the DEGs *via* R software.

### Construction of Co-expression network

The “WGCNA” package in R was introduced to construct the weighted gene co-expression network and obtain different gene modules by sample clustering (Langfelder and Horvath, 2008). Whole transcriptome expression was input to identified a co-expression module highly enriched for genes involved in neutrophil-related immune processes. After eliminating missing values, outliers, and redundant data, we found the optimal *β* value by adopting the pickSoftThreshold function of WGCNA to make the constructed network more consistent with scale-free network characteristics. The adjacency matrix was transformed into a topological overlap matrix. Clusters of correlated gene were classified into modules using topological overlap matrix (TOM) hierarchical clustering analysis and the Dynamic Tree Cut method. The correlation between gene modules and clinical status was calculated and the modules related to traits were identified after hierarchical clustering and Spearman correlation analyses. *p* values <0.05 and correlation coefficient (r) > 0.4 are statistically significant. Subsequently, we extracted genes in modules significantly associated with aSAH to perform in-depth analysis.

### Functional enrichment analysis and identification of hub genes

To explore the function and pathways of the genes in hub modules, the gene ontology (GO) and Kyoto Encyclopedia of Genes and Genomes (KEGG) pathway enrichment were conducted by using “ClusterProfiler” and “org.Hs.eg.db” packages in R (Yu et al., 2012). The overlapping genes between DEGs and the selected module were included for further analysis. We uploaded the overlapping genes into the STRING database (STRING; http://string-db.org) to construct protein-protein interaction (PPI) network (Szklarczyk et al., 2015). The minimum required interaction score is 0.7. Afterward, the network which removed disconnected nodes was visualized by Cytoscape software (Shannon et al., 2003). The cytoHubba plugin was applied to filter the hub genes which are highly interconnected in the PPI network.

### Connective map analysis

Connective Map (CMap) (Lamb et al., 2006) is a pharmacogenomics database based on the concept called “signature reversion” containing the expression profiles of cell lines before and after different drug treatments (Li et al., 2016). We use the eXtreme Sum (XSum) and a compromising parameter (topN = 200) to match neutrophil-related genes in aSAH and compound signatures in CMap, which has been proven to show optimal drug retrieval performance (Yang et al., 2022). Lower CMap scores generally correspond to higher reversal potency and greater potential for application.

### Identification of signature genes and construction of prediction model

To minimize the bias of statistical analysis, the two algorithms were performed to identify the signature genes of aSAH, including the least absolute shrinkage and selection operator (LASSO) and support vector machine recursive feature elimination (SVM-RFE). To obtain a more refined model, LASSO analysis constructed a penalty function of the overlapping genes mentioned above by adopting “glmnet” package in R. SVM-RFE is a powerful feature selection algorithm to avoid overfitting when the number of features is high. We performed “e1071” and “caret” packages to rank the features based on their weights and eliminate the feature with the lowest weight with five-fold cross-validation. The overlapping genes between above two algorithms are selected to construct a model and validated in the GSE73378, GSE15629, and GSE13353 datasets. Subsequently, Receiver operating characteristic (ROC) curve analysis evaluates the stability and sensitivity of the model in identifying aSAH.

### Gene set enrichment analysis

The gene set enrichment analysis (GSEA) on whole transcriptome expression was performed to identify the significant biological pathways post-aSAH. The gene sets of pathways were obtained from the Molecular Signatures Database (MSigDB) (Liberzon et al., 2011). The “clusterProfiler” R package was used for GSEA, genes were ranked according to the log fold change between aSAH versus control calculated by limma algorithm as previous suggested. No fold-change cutoff was set, all genes with *p* < 0.05 and FDR-adjusted *p*-value < 0.05 were considered significant. The GSEA enrichment plots were generated by R package “GseaVis” (https://github.com/junjunlab/GseaVis).

### Depiction of immune infiltration landscapes

CIBERSORT adopts a deconvolution algorithm to estimate the composition and abundance of immune cells in a mixture of cells based on gene expression data (Newman et al., 2015). We performed this bioinformatics algorithm which provides the expression date of 22 common immune infiltrating cells (LM22) to compare the fraction of 22 immune cells between the aSAH and the control samples. Likewise, the single-sample gene-set enrichment analysis (ssGSEA) was introduced to depict the immune infiltration landscape and enrichment of immune cells and immune function. Additionally, the relationship between identified core diagnostic genes and infiltrating immune cells was evaluated using Spearman correlation analysis. The resulting associations were visualized by the “ggplot2” package in R software.

### Statistical analysis

All statistical analyses were conducted using R statistics software (version 4.1.1). The Wilcoxon test was performed for inter-group comparisons. The correlation between the variables was determined by Pearson’s correlation test. *p* < 0.05 was considered to be statistically significant.

## Results

### Identification of differential expressed genes between aneurysmal subarachnoid hemorrhage and normal samples

After comparing 43 aSAH samples with 18 normal samples in aSAH dataset GSE 36791, 602 DEGs were obtained (including 315 up-regulated DEGs and 287 down-regulated DEGs) under the threshold of adjusted *p*-value < 0.05 and | log2 FC | >0.5. The identified DEGs were visualized by the volcano plot and heatmap (Supplementary Figure S1).

### Construction of weighted gene Co-expression network and enrichment analysis

**FIGURE 2:**
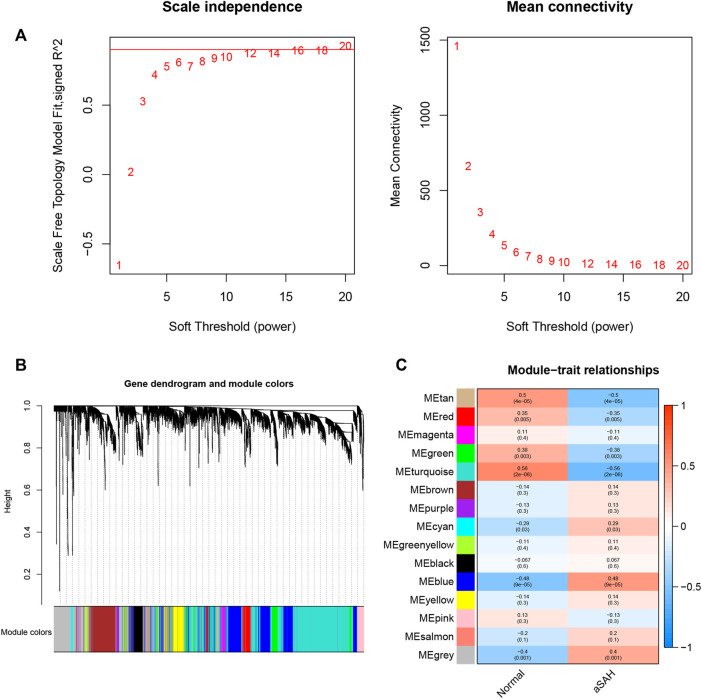
WGCNA revealed gene co-expression networks in the whole peripheral blood of aSAH patients. **(A)** Determination of soft-thresholding power. The red line indicates where the correlation coefficient is 0.9, and the corresponding soft-thresholding power is 12. **(B)**.Hierarchical cluster tree showing fifteen modules of co-expressed genes identified by WGCNA. Each branch in the figure represents one gene, and every color below represents one co-expression module. **(C)** Relationships of consensus modules with samples.

Whole transcriptome expression was applied to identified modules of highly correlated gene. Before building the weighted gene co-expression network using the package “WGCNA” in R software, we performed a crucial step that search the soft-thresholding power (Figure 2A). When the soft threshold power was set as 12, the scale-free topology fit index reached 0.9. The dynamic branch cut method was adopted to identify a total of 15 modules in which the number of genes is not ＜30 (Figure 2B). As shown in Figure 2C, three modules (blue: cor = 0.48, p = 9e-05; turquoise: cor = −0.56, p = 2e-06; tan: cor = −0.5, p = 4e-05) which demonstrated remarkable correction with the occurrence of aSAH was considered as the candidate modules for further analysis.

### Gene ontology and kyoto encyclopedia of genes and genomes enrichment analysis and protein-protein interaction network analysis

**FIGURE 3:**
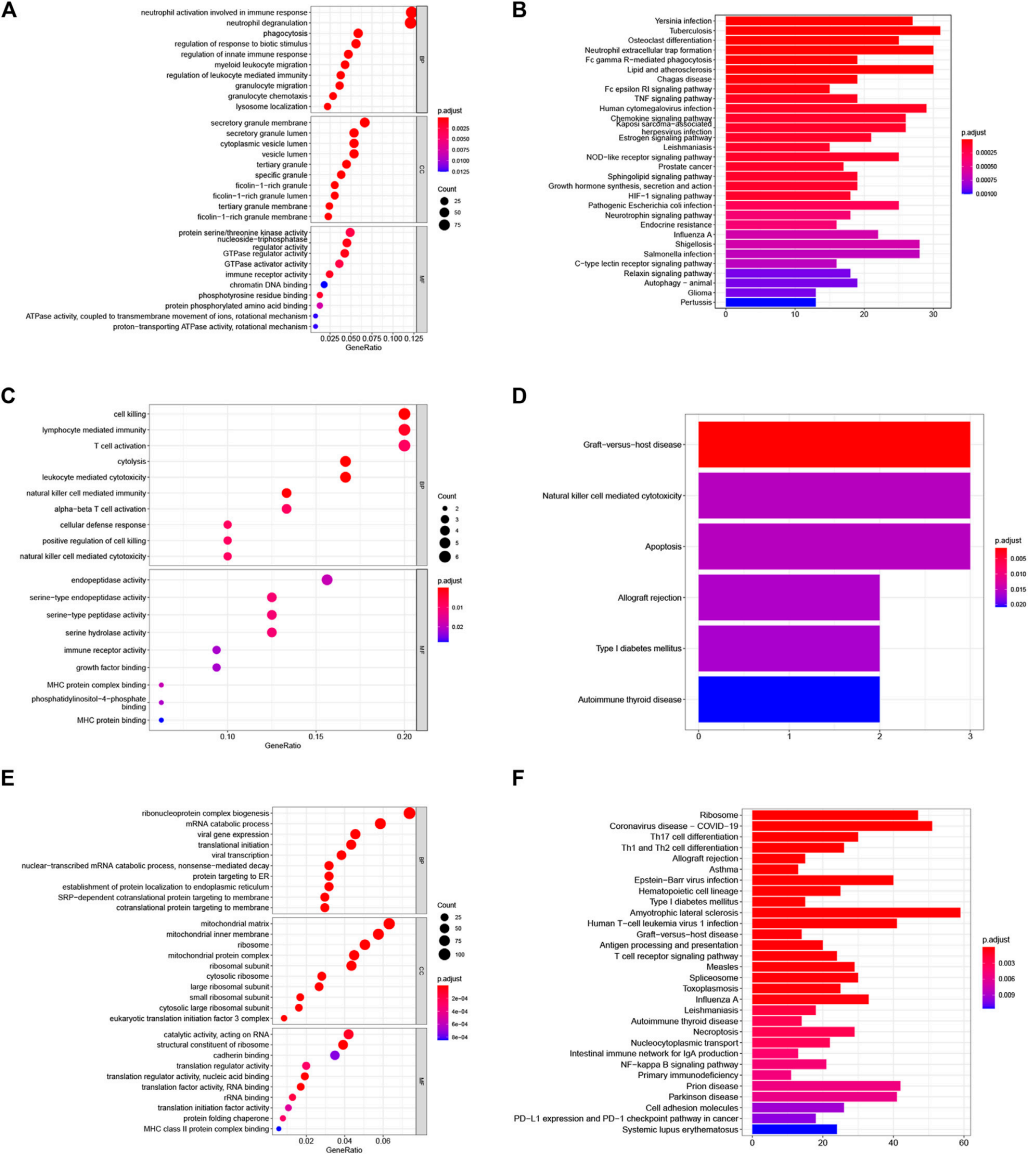
Functional enrichment analysis of the aSAH-related module. **(A,B)** The GO and KEGG analysis of the genes in the blue module. **(C,D)** The GO and KEGG analysis of the genes in the tan module. **(E,F)** The GO and KEGG analysis of the genes in the turquoise module.

To explore the potential biological features of aSAH-associated co-expression modules, we performed the GO and KEGG enrichment analysis. Due to the key role of the activation of the immune response in the pathophysiology of SAH, we focused on immune-related modules. In terms of biological processes (BP), the blue module was primarily related to neutrophil activation involved in immune response, neutrophil degranulation, granulocyte migration, myeloid leukocyte migration, phagocytosis, granulocyte chemotaxis, regulation of leukocyte mediated immunity, regulation of innate immune response, etc. The tan module was markedly associated with cytolysis, cell killing, leukocyte mediated cytotoxicity, natural killer cell-mediated immunity, lymphocyte-mediated immunity, T cell activation, natural killer cell-mediated cytotoxicity and so on. The KEGG pathways in the blue module are enriched in tuberculosis, neutrophil extracellular trap formation, Fc gamma R-mediated phagocytosis, lipid and atherosclerosis, chemokine signaling pathway, NOD-like receptor signaling pathway, HIF-1 signaling pathway and so on. The details of GO and KEGG analysis of three aSAH-associated modules are shown in Figure 3. Taken together, these findings strongly suggest that the immune response plays an essential role in aSAH.

**FIGURE 4:**
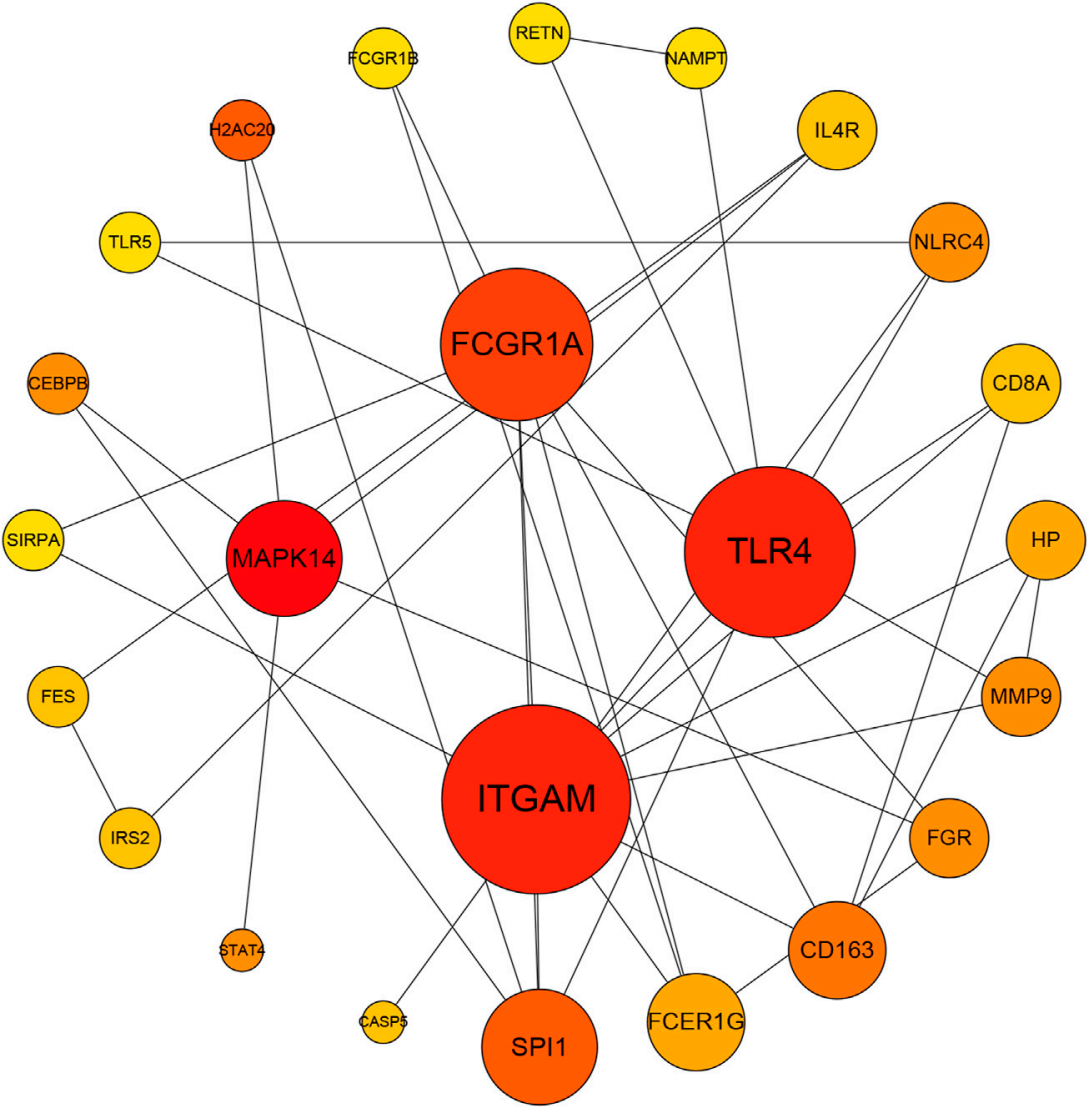
The Protein-protein interaction network of the overlapping genes between blue module and DEGs by Degree analysis. MAPK14, ITGAM, TLR4, and FCGR1A were calculated as the top four hub genes.

The blue module was associated with neutrophil-related immune responses according to its GO annotations. Considering that the blue module-related immune response is significantly positive associated with aSAH, we selected it for subsequent analysis. The intersection of the genes in blue modules and DEGs including 197genes was selected to construct a PPI network comprised of 186 nodes and 95 edges. The top four highly connective genes were screened by the degree algorithm of CytoHubba plugin, including MAPK14, ITGAM, TLR4, and FCGR1A (Figure 4).

### Drug targeting based on the gene expression in neutrophil-related module

**FIGURE 5:**
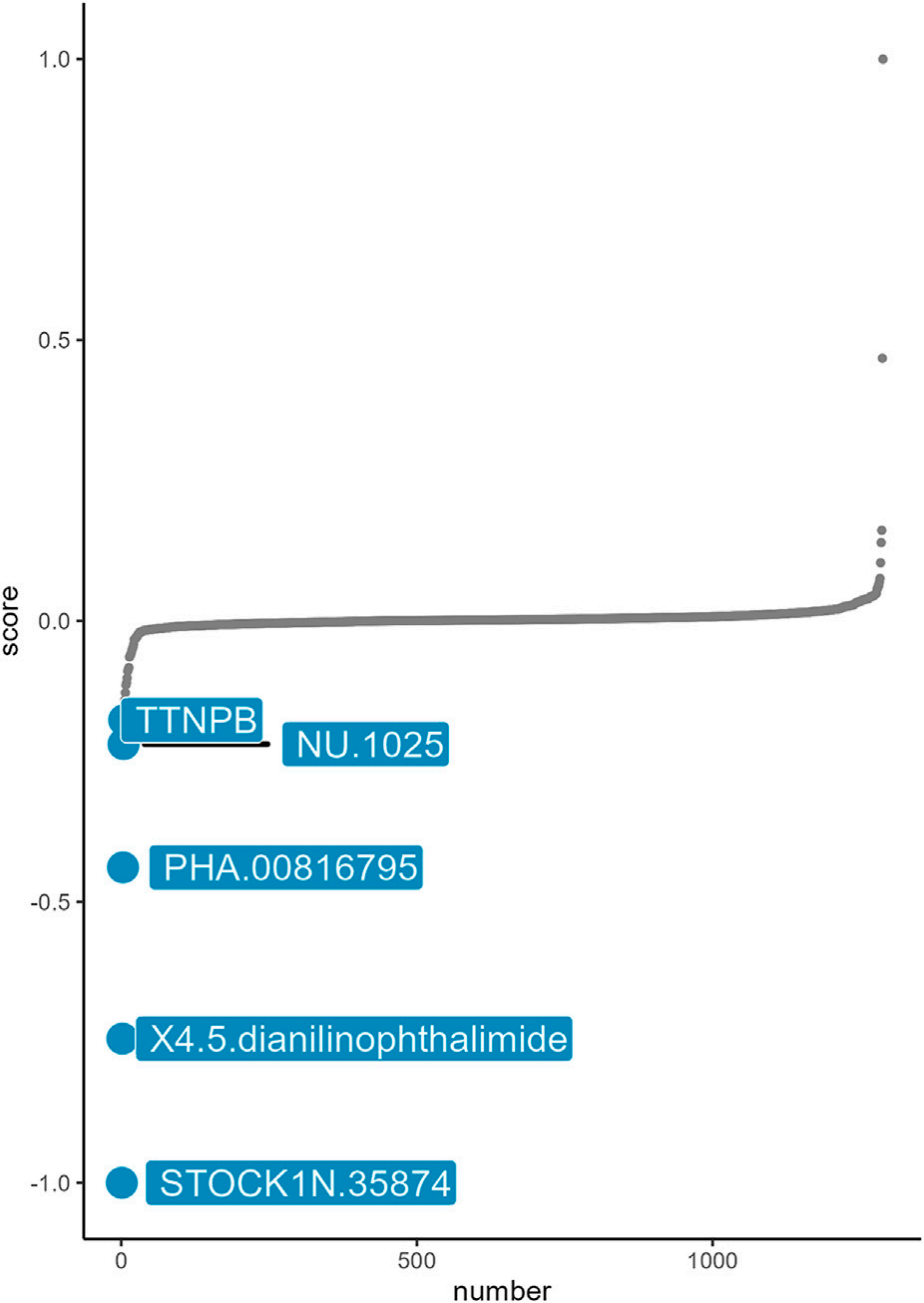
Small molecular compounds identified by connectivity map.

Based on the basic concept of signature reversal, we use CMap analysis to identify the drugs that target neutrophil-related immune inflammation with the ability to reverse neutrophil-associated gene expression pattern. CMap analysis identified five small-molecule compounds including STOCK1N-35874, X4.5. dianilinophthalimide, PHA.000816795, NU.1025, TTNPB, which could be developed as potential targeted drugs for the treatment of aSAH (Figure 5). STOCK1N-35874 had a lowest CMap score indicated highest reversal potency.

### Identification and verification of biomarkers

**FIGURE 6:**
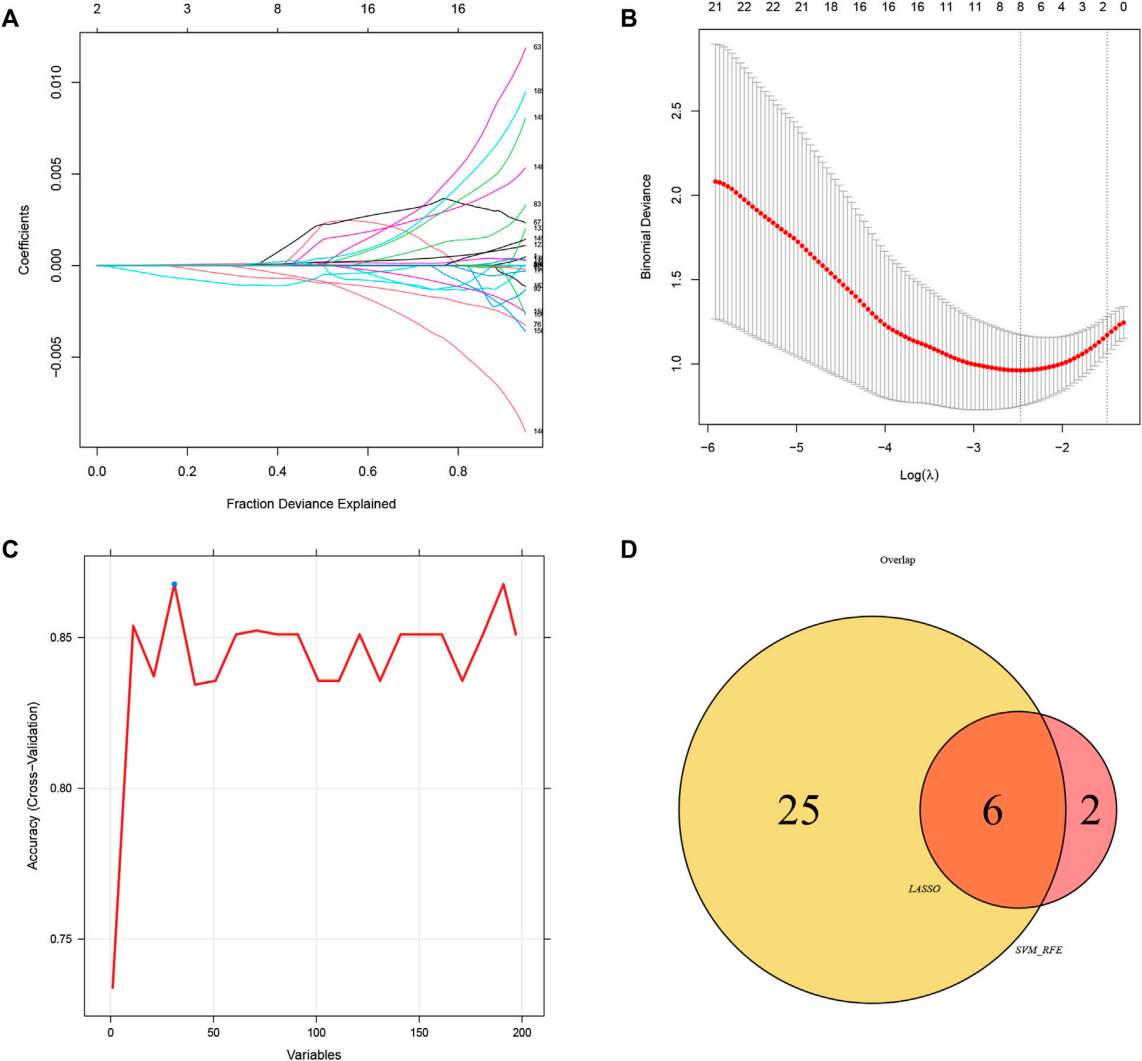
Identification of diagnostic biomarkers *via* comprehensive strategy. **(A,B)** Identification of biomarkers using the LASSO regression algorithm. **(C)** Based on the SVM-RFE algorithm to screen biomarkers. **(D)** Venn diagram demonstrate six markers obtained by the LASSO regression algorithm and SVM-RFE

**FIGURE 7:**
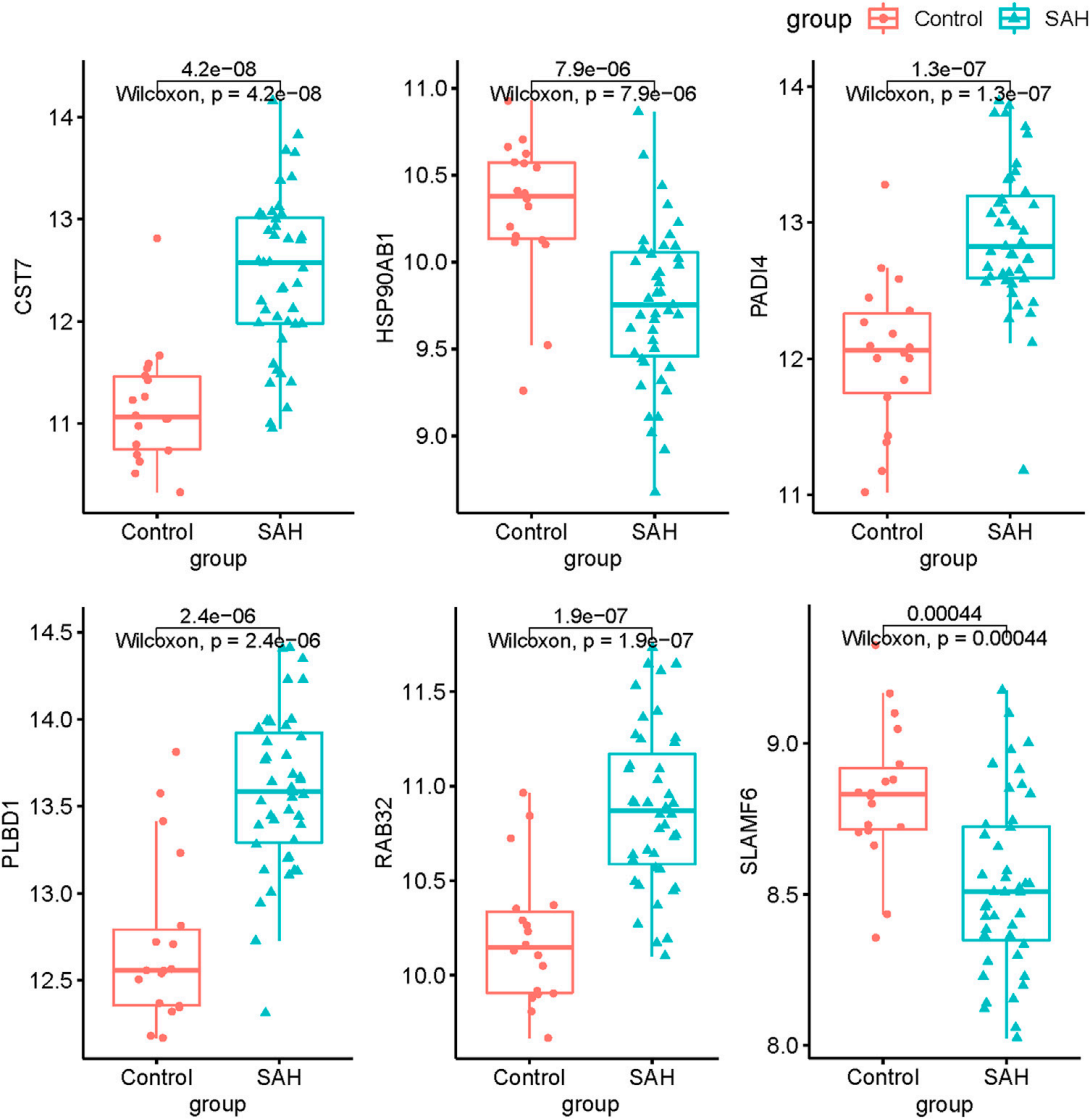
The expression levels of signature genes in GSE3691.

**FIGURE 8:**
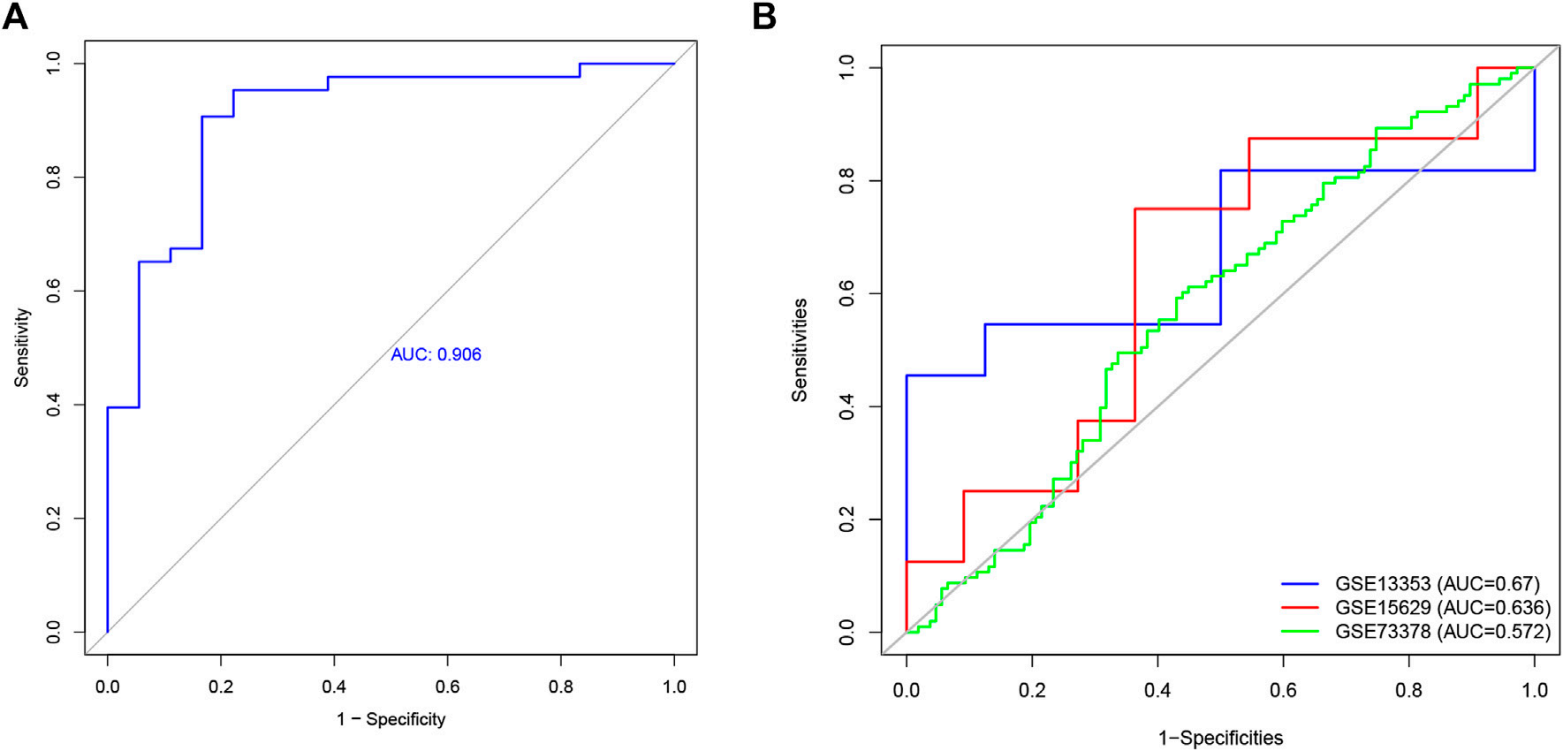
The receiver operating characteristic (ROC) curve of the diagnostic effectiveness of the six diagnostic markers. **(A)** ROC curve of six genes in GSE36791. **(B)** Validation the diagnostic value in GSE13353, GSE15629, and GSE 73378 dataset.

To explore the feature diagnostic markers, we extracted the 197 genes obtained from the intersection between the blue module and DEGs. A total of 25 genes were screened as feature biomarkers by the LASSO regression analysis.8 genes were identified as vital biomarkers in accordance with SVM-RFE analysis (Table 1). The overlapping genes including CST7, HSP90AB1, PADI4, PLBD1, RAB32, and SLAMF6 were selected as diagnostic biomarkers by the above two algorithms (Figure 6). The expression values of core genes were compared between patients with post-aSAH and patients with non-aSAH (Wilcoxon rank-sum test). As shown in Figure 7, the expression levels of CST7, PADI4, PLBD1, and RAB32 were significantly upregulated in aSAH, then HSP90AB1 and SLAMF6 were lower than those in healthy controls. Additionally, the AUC of the training set based on the above six biomarkers was 0.906, indicating a robust discrimination ability of the constructed model. Furthermore, we confirmed the predictive value of the model in validation sets, with the AUC of 0.67 in GSE13353, AUC of 0.636 in GSE15629, and AUC of 0.572 in GSE73378 (Figure 8).

**TABLE 1:** The key gene list of LASSO and SVM-RFE analysis.

Analysis	Genes
LASSO	CST7, GPER1, HSP90AB1, PADI4, PLBD1, RAB32, RPL36P14, SLAMF6
SVM-RFE	HK3, PGD, PLBD1, PADI4, DYSF, CST7, SLAMF6, RAB32, GYG1, MSRB1, MTHFS, TSPO, SRPK1, USB1, FUT7, IRAK3, HSP90AB1, UBTD1, MMP9, TLR5, ALPL, SLC26A8, WSB1, KIF1B, APMAP, SLC25A44, GTF2IP20, S100A12, IL18RAP, UBE2J1, XPO6

The six genes highlighted in bold are the overlap of the results of the LASSO, and SVM-RFE, analysis.

### Signaling pathways involved in aneurysmal subarachnoid hemorrhage

**FIGURE 9:**
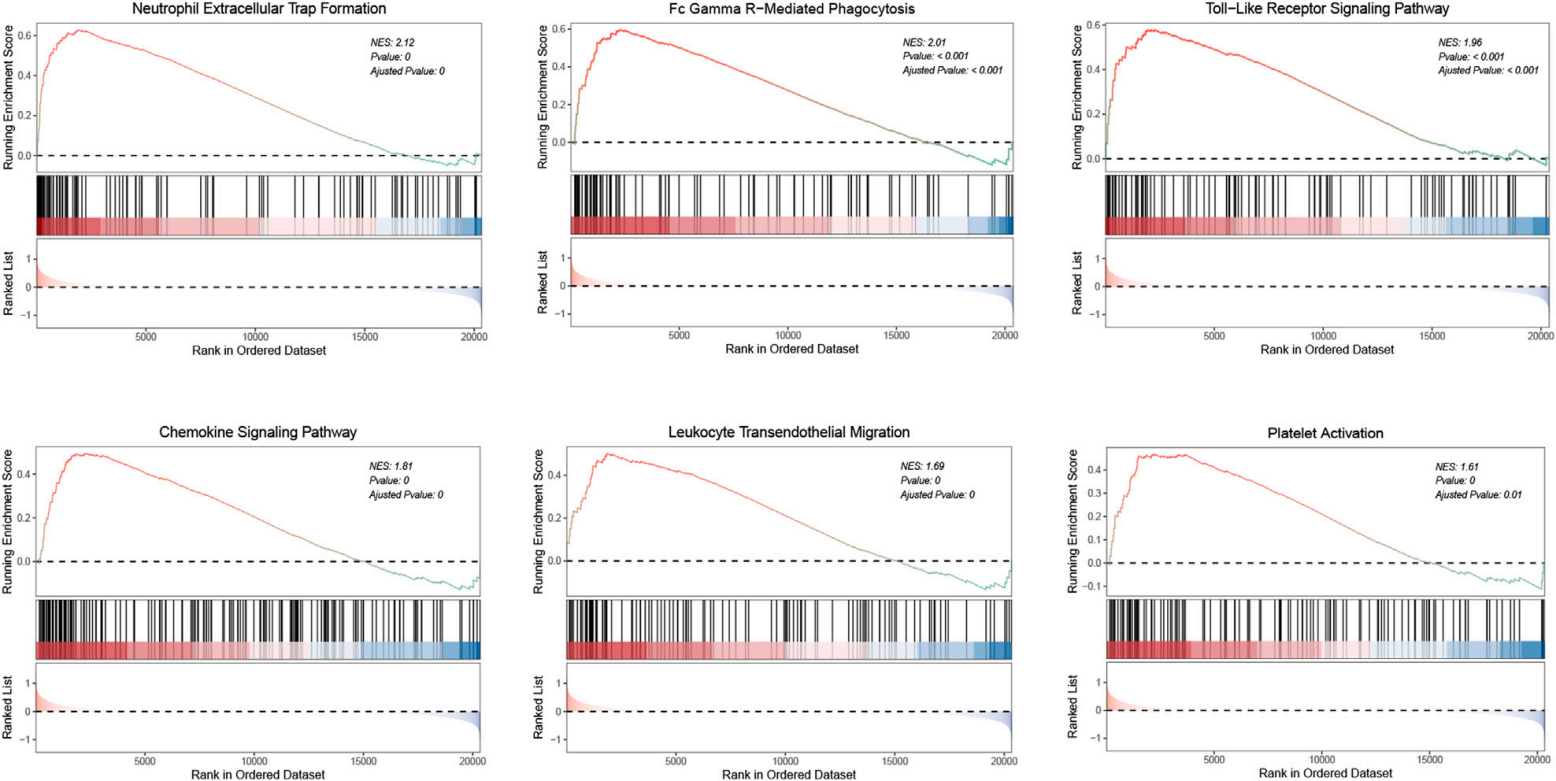
GSEA analysis illustrates the activated pathways in aSAH. The normalized enrichment scores (NES) and *p* values are indicated in each plot.

We conducted GSEA on whole-genome data to further illustrate the holonomic biological functions of the transcriptome and evaluated signaling pathways involved in the characteristic genes and hub genes. The GSEA results revealed that the following pathways were significantly upregulated in aSAH group: neutrophil extracellular trap formation (NES = 2.12), Fc gamma R-mediated phagocytosis (NES = 2.01), toll-like receptor signaling pathway (NES = 1.96), chemokine signaling pathway (NES = 1.81), leukocyte transendothelial migration (NES = 1.69), and platelet activation (NES = 1.61) (Figure 9). It was consistent with previous findings regarding GO and KEGG enrichment analysis. Therefore, these results may provide insights into the cellular biological effect of neutrophils, which could promote the inflammatory response after aSAH by releasing the neutrophil extracellular traps (NETs).

### Immune infiltration analyses

**FIGURE 10:**
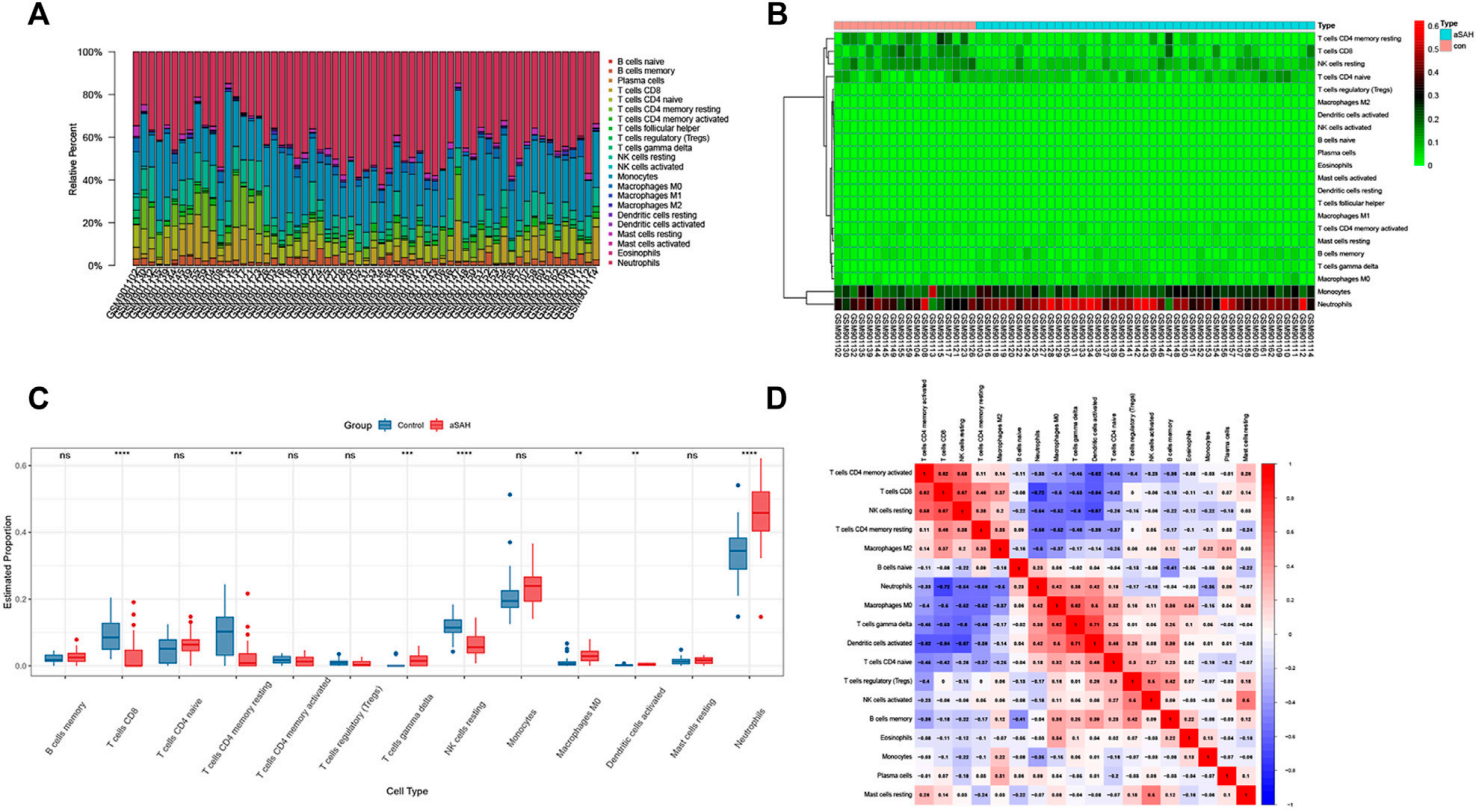
Comparisons of 22 important immune fractions by CIBERSORT. **(A)** The Specific 22 immune fractions represented by various colors in each sample were shown in the barplot. **(B)** Heatmap of correlation in 22 types of immune cells. **(C)** Box plot of the proportion of 22 types of immune cells for aSAH versus control. Immune cells that were expressed in less than half the samples were excluded. **(D)** Correlation matrix of all 22 immune cell subtype compositions. Red represents a positive correlation, and blue represents a negative correlation.

**FIGURE 11:**
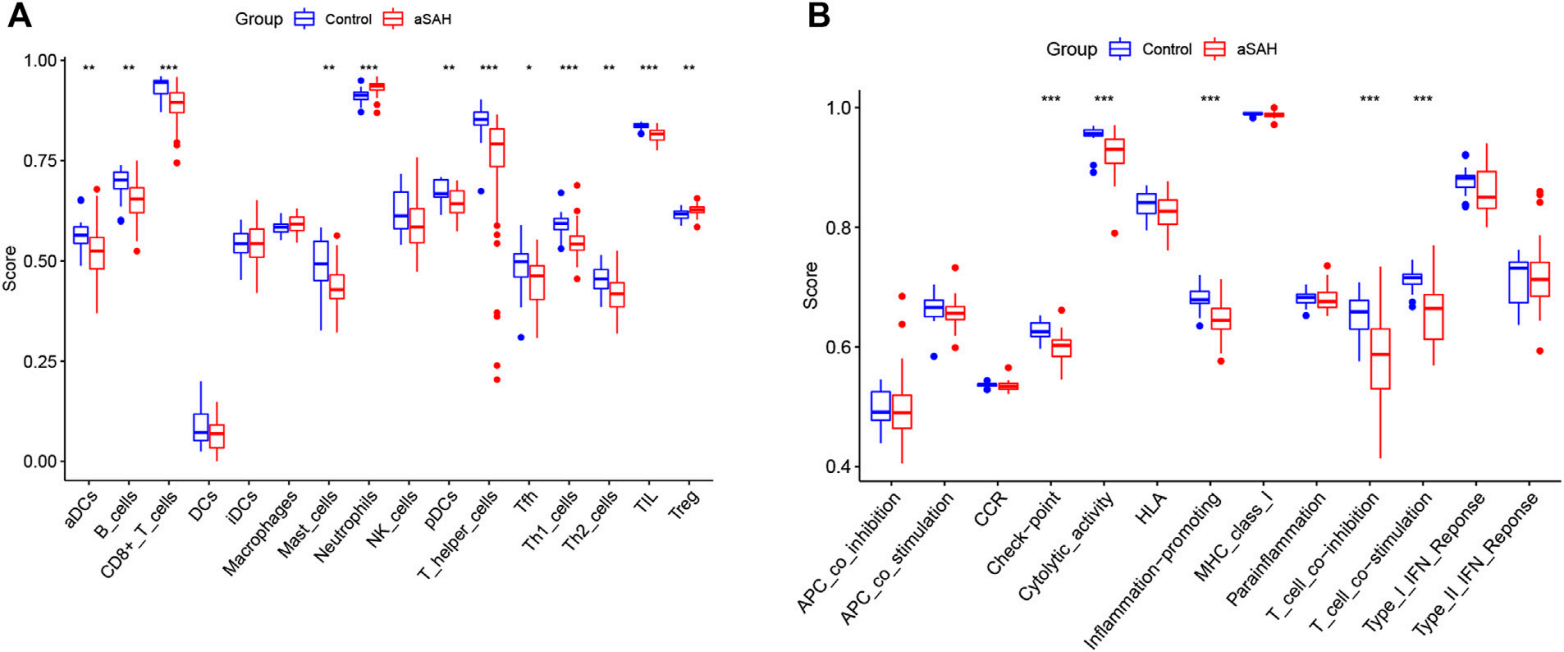
Boxplot of differential immune cells **(A)** and immune features **(B)** between control and aSAH group in GSE36791; *p < 0.05; **p < 0.01; ***p < 0.001.

We analyzed the overall proportion pattern of 22 immune cells in aSAH and control samples to explore the immune microenvironment in aSAH by the CIBERSORT algorithm (Figure 10). The distribution of 22 immune cell types differed significantly in each sample, but the neutrophils dominated the enriched proportion of all samples (Figure 10A). Furthermore, compared with the control samples, the distribution of neutrophils (*p* < 0.001) was highly in aSAH groups which indicated that the neutrophils play vital roles in potential immunoregulatory mechanisms of aSAH pathogenesis (Figures 10B,C). The correlation heatmap of the immune cells revealed that CD8 T cells had negative correlation with neutrophils (−0.72) (Figure 10D). To further investigate differences in the immune function between aSAH samples and control samples, we performed the ssGSEA analysis. Compared to the control group in the GSE36791, neutrophils were similarly elevated in aSAH group, and various antigen presentation processes consisting of checkpoint, cytolytic activity, inflammation promoting, T cell co-inhibition, T cell co-stimulation were found to be remarkably weaker (Figure 11).

### Correlation analysis between the six biomarkers and immune cells

**FIGURE 12:**
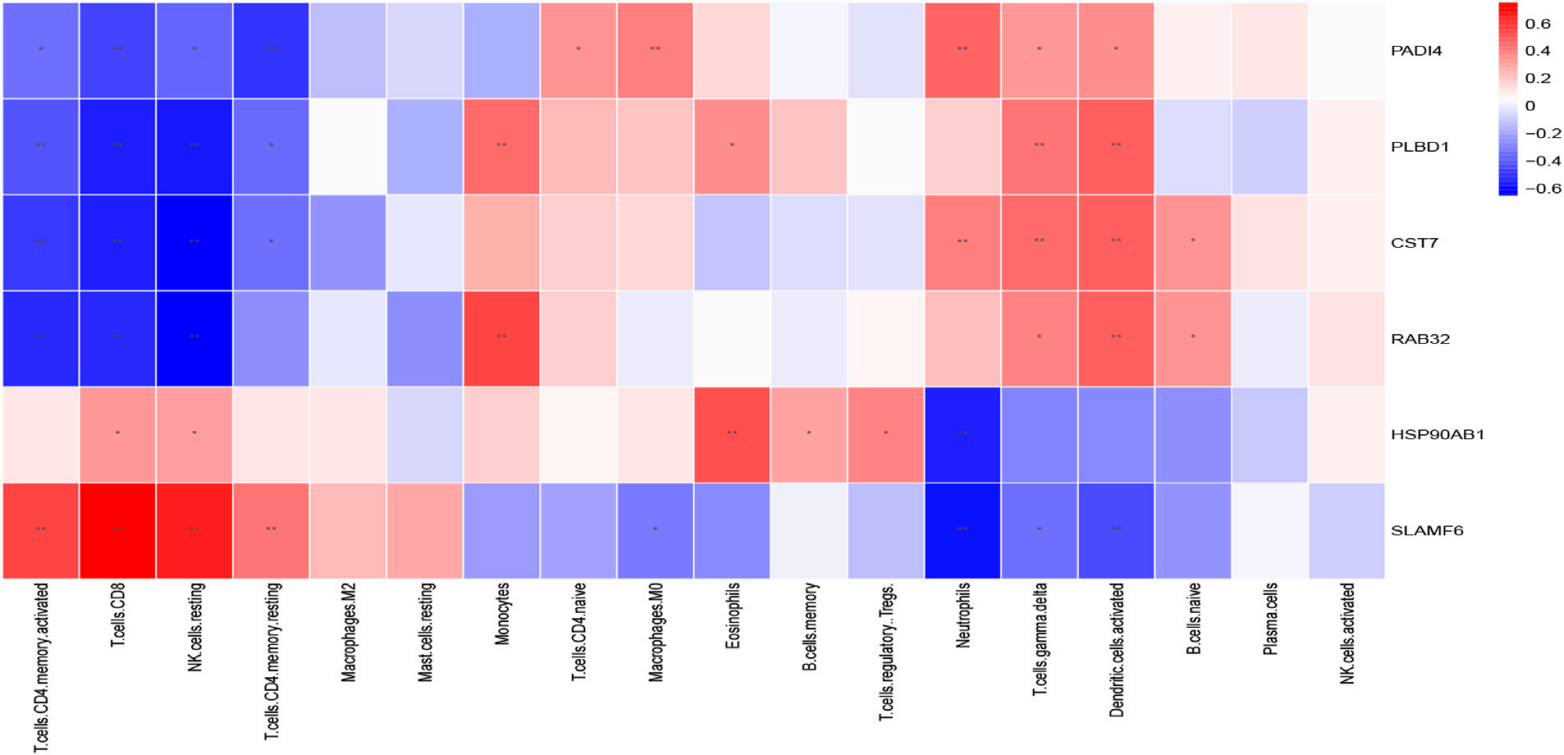
The correlation between the immune cells and diagnostic biomarkers; *p < 0.05; **p < 0.01.

The Spearman correlation heatmap described the correlation of the hub genes related to immune cells (Figure 12). The results displayed that PADI4 had a positive correlation with neutrophils, macrophages (M0), naive CD4(+) T cells, gamma delta T cells and activated dendritic cells. PLBD1 had a positive correlation with gamma delta T cells, activated dendritic cells, monocytes and eosinophils. CST7 had a positive correlation with neutrophils, gamma delta T cells, activated dendritic cells and naive B cells. PAD4, PLBD1 and CST7 had negative correlated with CD8(+) T cells, resting memory CD4(+) T cells, activated memory CD4(+) T cells and resting natural killer (NK) cells. RAB32 had a positive correlation with monocytes, activated dendritic cells, gamma delta T cells and naive B cells, and negative correlation with CD8(+) T cells, activated memory CD4(+) T cells and resting natural killer (NK) cells. HSP90AB1 had a positive correlation with eosinophils memory B cells, Tregs, CD8(+) T cells and resting natural killer (NK) cells, and negative correlation with neutrophils. SLAMF6 had a positive correlation with CD8(+) T cells, resting memory CD4(+) T cells, activated memory CD4(+) T cells and resting natural killer (NK) cells, and negative correlation with neutrophils, activated dendritic cells, gamma delta T cells and macrophages (M0).

## Discussion

Nowadays, systemic inflammation and immune dysregulations have become a hot topic under discussion which is closely associated with aSAH complications and poor prognosis. Tracing the potential cellular and molecular mediators of immune function subsequent to aSAH may be crucial in the discovery of diagnostic and therapeutic targets. After aSAH, recruited peripheral immune cells in response to increased expression of chemokines can enter the CNS which may correlate with the modulating of microglial functions. The screening of potential genes after aSAH and revealing their biological pathways is considered an effective strategy for predictive diagnosis and treatment of the disease. However, no immune-related genes have been studied in aSAH. In our study, we acquired publicly available microarray data and applied integrating bioinformatic analysis to screen the DEGs genes between aSAH and non-aSAH control group. We focused on immune-related function in these genes and found that CST7, HSP90AB1, PADI4, PLBD1, RAB32, and SLAMF6 may play crucial roles in the diagnosis of aSAH. Particularly, PADI4 is strongly correlated with neutrophils and may be a potential biomarker for aSAH.

We explored gene correlation at the RNA level by using WGCNA to provide key gene modules showing co-expression patterns. Here, we revealed that neutrophil-related immune responses enriched in the blue module were notably upregulated in patients with aSAH, while lymphocyte-related genes enriched in the tan module were significantly downregulated by GO and KEGG enrichment analysis. Accordingly, aSAH achieved robust immune activating process and immune cell involvement. To further investigate the impact of immune cell infiltration in aSAH, this study applied CIBERSORT and ssGSEA to conduct a comprehensive evaluation of the immune infiltration process in aSAH. The infiltration of neutrophils increased and the depression of lymphocyte response that probably associated with the progression of aSAH. Besides, genes encoding neutrophil cell surface markers, such as ITGAM (CD11b), FCGR1 (CD64), were identified as the hub genes, indicating the activation status of neutrophils. These implied that targeting neutrophils potentially indicates a therapeutic window to attenuate neuroinflammatory after SAH.

We further detected the neutrophils’ potential involvement in pathophysiological processes in aSAH, MAPK14, ITGAM, TLR4, and FCGR1A shared highly centrality with the PPI network and CytoHubba. Toll-like receptors (TLRs) widely expressed in the central nervous system (CNS) are well-established mediators to upregulate inflammation (Takeuchi and Akira, 2010). TLR4, a critical member of the TLR family, has long been thought to play a major role in the immune response after aSAH (Lu et al., 2018; Gao et al., 2019; Karimy et al., 2020). TLR4 can be activated by many endogenous ligands having DAMPs including heme, oxyhemoglobin, methemoglobin, fibrinogen, peroxiredoxin 2, high mobility group box 1 protein (HMGB1), the matricellular protein tenascin-C and galectin-3, all of which are abundantly produced after SAH (Hanafy, 2013; Kwon et al., 2015; Kawakita et al., 2017; Lu et al., 2018; Nishikawa et al., 2018; Zhang et al., 2019), these lead to the activation of downstream inflammatory signaling cascades, including the NF-κB, MyD88/TRIF, and mitogen-activated protein kinase (MAPK) pathways (Sasaki et al., 2004; Suzuki et al., 2010b; Suzuki et al., 2010c). To our knowledge, compared to NF-κB signaling pathway, the TLR4/MAPK pathway has received scant attention in studies of early brain injury after aSAH. Some studies have linked MAPK to cytokines activation in response to inflammation in post-aSAH (Kusaka et al., 2004; Vikman et al., 2007; Maddahi et al., 2012; Chen et al., 2013; Guo et al., 2016). In pro-inflammatory mediators induced by TLR4, metalloproteinase-9 (MMP-9) can degrade interendothelial tight junction proteins such as ZO-1, and thereby causing BBB disruption and neurological impairment in mice (Suzuki et al., 2010a; Rubio-Araiz et al., 2017). Some studies have reported elevated activity of MMP-9 in brain tissue, serum, and cerebrospinal fluid after SAH (Egashira et al., 2015; Okada et al., 2021; Yu et al., 2021). A study concluded that post-SAH induction of tenascin-C disrupted the BBB by triggering three MAPK subgroups (JNK, ERK1/2, and p38) (Fujimoto et al., 2016). Okada et al. found that TLR4 antagonists alleviated post-aSAH BBB disruption and cerebral edema *via* MMP-9 downregulation, and also demonstrated that the TLR4/MAPK pathway is involved in brain damage in endovascular perforation SAH mice (Okada et al., 2019). Consistently, the constructing PPI network in our result provides evidence that MAPK14 (p38α) activation was shown to make greater contributions to TLR4-mediated MMP-9-induced post-aSAH brain injury.

To further investigate the potential utilization of genes in diagnostic development for aSAH, we perform classification by the machine learning algorithm. Our analysis demonstrated good diagnostic efficiency. The diagnostic biomarkers, CST7, HSP90AB1, PADI4, PLBD1, RAB32, and SLAMF6, identified by machine-learning strategies may provide new insight into the mechanism of inflammatory responses in aSAH. The constructed model consisting of above six signature genes showed lower discrimination power in GSE73378 which included the SAH survivors who had the last episode of aSAH at least 2 years and control patients. It can be inferred that the present signature genes may be unique for acute aSAH.

Peptidylarginine deiminase 4 (PADI4) is primarily expressed in neutrophils, encoding peptidylarginine deiminase 4 (PAD4) which catalyzes the deimination of histone arginine (Arg) residues to citrulline (Cit). PAD4 triggered the formation of neutrophil extracellular traps (NETs) by inducing chromatin decondensation. Intriguingly, neutrophil extracellular trap formation achieved robust immune acting process among the identified KEGG analysis, and PADI4 suggested a positive correlation with neutrophils. NETs are released by highly activated neutrophils, which are extracellular DNA network structures decorated with cytotoxic enzymes and histones (Sørensen and Borregaard, 2016). Besides trapping and neutralizing pathogens in the innate immune responses, NETs also mediate sterile inflammation including in the development of brain injury. Recent studies found that NETs involve in the pathophysiology of stroke and are related to severity and mortality (Vallés et al., 2017; Kang et al., 2020). NETs contribute to impair revascularization and vascular remodeling and BBB damage and exacerbate brain injury in a variety of central nervous system diseases (Thanabalasuriar et al., 2019; Kang et al., 2020; Vaibhav et al., 2020; Wang et al., 2021). Recent animal experiments have confirmed the formation of NETs in the pathological progression after SAH. DNase I (digestion of NETs), GSK484 (inhibitor of PAD1), and anti-Ly6G antibody (inhibitor of neutrophil) can alleviate brain edema and attenuate neurological dysfunction, even including neurogenic pulmonary edema post-SAH. In addition, NETs may involve in the progress of the pro-inflammatory subtype microglia activation and inflammatory cytokine production, thus exacerbating subsequent brain damage (Zeng et al., 2022). Currently, the signaling pathways involved in NETs formation are not well identified. A recent study demonstrated that TAK1, p38 MAPK, and MEK affected NET generation. Moreover, the activated neutrophils could release endogenous factors identified as peptides or proteins, which promoted NETs induction. Additionally, integrin alpha M precursor (ITGAM), Phospholipase B Domain Containing 1 (PLBD1) and MMP-9 featured at least a 2-fold induction by mass spectrometry proteomics analysis (Tatsiy et al., 2021). Thus, it is reasonable to hypothesize that post-SAH TLR4-mediated BBB disruption involves activation of MAPKs, generation of NETs and upregulation of MMP-9.

Growing evidence demonstrates that microthrombi contribute to infarction and the development of delayed cerebral ischemia (DCI) and neurological deficit after aSAH (Dienel et al., 2020). Along with platelet activation, upregulation of P-selection and von-Willebrand factor (vWF) after aSAH have been reported to participate in platelet-leukocyte-endothelial cell interactions (Ishikawa et al., 2009; Frontera et al., 2012; Dienel et al., 2020). The platelet antagonist (P-selectin antibody) and ADAMTS13 (vWF-cleaving metalloprotease) can reduce leukocyte adhesion (Chauhan et al., 2008). Besides, NETs can be driven by exposure to activated platelets which may be responsible for immunothrombosis (Maugeri et al., 2014). The active TLR4 on platelets reportedly induced platelet binding to adherent neutrophils, leading to the release of NETs in sepsis (Clark et al., 2007). As well, NETs can regulate coagulation by being involved in fibrin polymerization, vWF binding, platelet recruitment, and so on (Swystun and Liaw, 2016). PAD4 also prevented the removal of vWF in the vessel wall, thereby enhancing NETs attachment and thrombosis (Fuchs et al., 2010). Besides, neutrophil surface expression of CD11b (integrin αM, ITGAM) was considered as the hallmark of secretory vesicle degranulation (Naegelen et al., 2015; Mauler et al., 2019). In acute ischemic stroke, highly activated circulating neutrophils were characterized by higher CD11b expression at the cell surface (Weisenburger-Lile et al., 2019). In addition, leukocyte β2-integrin Mac-1 (also known as αMβ2, CD11b/CD18) involved in mediating leukocyte-platelet interactions may be an antithrombotic target (Wang et al., 2017; Plow et al., 2018). Overall, a vicious circle leads to pathological thrombus formation may be triggered. Activation of neutrophils and NETs-related immunothrombosis may offer insight into the mechanism of microthrombi in aSAH patients and suggest that the NETs could be an antithrombotic target, which still requires further investigation.

CMap analysis was conducted to identify small molecule drugs with potential efficacy in neutrophil-associated inflammation post-aSAH including STOCK1N-35874, 4,5-dianilinophthalimide and so on. STOCK1N-35874 is a cytotoxic quinoline alkaloid that inhibits the DNA enzyme topoisomerase (Wink, 2007), which may attenuate the inflammatory response through disrupting DNA in the NET structure. 4,5-dianilinophthalimide (DAPH) is a selective inhibitor of epidermal growth factor receptor (EGFR) kinase (Trinks et al., 1994). It has been reported that EGFR inhibitors could bring anti-vasospastic effect after experimental SAH (Nakano et al., 2019). There have been few literature reports concerning the interaction between these drugs and aSAH. Our work may help to provide new strategies for the drug treatment of aSAH. Our study had several limitations that should be noted. First, the main limiting factors were that the sample size was small and imbalanced, which required further real-world studies to verify our results. Although we have included multiple datasets for verification, *in vivo* or *in vitro* experiments with large samples are needed to further confirm our conclusions. Second, we did not explore the correlation between circulating neutrophils and prognostic outcomes owing to the lack of complete clinical information, and whether the results here have clinical applicability should receive in-depth verification in clinical research with large samples. Besides, the results could also be validated in human aSAH brain specimens. Finally, our study provided guidance on the role of circulating neutrophils in aSAH, and attention should be given to the dynamic changes in immune cell function and immune inflammation in different stages post-aSAH in further research.

## Conclusion

In summary, this bioinformatic study enhances the understanding of systemic inflammation in aSAH, helping to elucidate the molecular basis of neutrophil-related immune response pathways. Our findings give clues that NETs observed in aSAH may offer avenues for therapeutic intervention to reduce neuroinflammation involved in neuron damage, BBB disruption and microthrombi formation.

## Data Availability

Publicly available datasets were analyzed in this study. This data can be found here: All the raw data used in this study are derived from the public GEO data portal (https://www.ncbi.nlm.nih.gov/geo/; Accession numbers: GSE36791, GSE13353, GSE15629, and GSE73378).

## References

[B1] Atangana E. Schneider U. C. Blecharz K. Magrini S. Wagner J. Nieminen-Kelhä M. (2017). Intravascular inflammation triggers intracerebral activated microglia and contributes to secondary brain injury after experimental subarachnoid hemorrhage (eSAH). Transl. Stroke Res. 8 (2), 144–156. 10.1007/s12975-016-0485-3 27477569

[B2] Chaudhry S. R. Güresir A. Stoffel-Wagner B. Fimmers R. Kinfe T. M. Dietrich D. (2018a). Systemic high-mobility group box-1: A novel predictive biomarker for cerebral vasospasm in aneurysmal subarachnoid hemorrhage. Crit. Care Med. 46 (11), e1023–e1028. 10.1097/ccm.0000000000003319 30028365

[B3] Chaudhry S. R. Hafez A. Rezai Jahromi B. Kinfe T. M. Lamprecht A. Niemelä M. (2018b). Role of damage associated molecular pattern molecules (DAMPs) in aneurysmal subarachnoid hemorrhage (aSAH). Int. J. Mol. Sci. 19 (7). 10.3390/ijms19072035 PMC6073937 30011792

[B4] Chauhan A. K. Kisucka J. Brill A. Walsh M. T. Scheiflinger F. Wagner D. D. (2008). ADAMTS13: A new link between thrombosis and inflammation. J. Exp. Med. 205 (9), 2065–2074. 10.1084/jem.20080130 18695007 PMC2526201

[B5] Chen S. Ma Q. Krafft P. R. Chen Y. Tang J. Zhang J. (2013). P2X7 receptor antagonism inhibits p38 mitogen-activated protein kinase activation and ameliorates neuronal apoptosis after subarachnoid hemorrhage in rats. Crit. Care Med. 41 (12), e466–e474. 10.1097/CCM.0b013e31829a8246 23963136 PMC3841260

[B6] Chou S. H. Feske S. K. Simmons S. L. Konigsberg R. G. Orzell S. C. Marckmann A. (2011). Elevated peripheral neutrophils and matrix metalloproteinase 9 as biomarkers of functional outcome following subarachnoid hemorrhage. Transl. Stroke Res. 2 (4), 600–607. 10.1007/s12975-011-0117-x 22207885 PMC3236293

[B7] Clark S. R. Ma A. C. Tavener S. A. McDonald B. Goodarzi Z. Kelly M. M. (2007). Platelet TLR4 activates neutrophil extracellular traps to ensnare bacteria in septic blood. Nat. Med. 13 (4), 463–469. 10.1038/nm1565 17384648

[B8] Dienel A. Ammassam Veettil R. Hong S. H. Matsumura K. Kumar T. P. Yan Y. (2020). Microthrombi correlates with infarction and delayed neurological deficits after subarachnoid hemorrhage in mice. Stroke 51 (7), 2249–2254. 10.1161/strokeaha.120.029753 32539672 PMC7316137

[B9] Egashira Y. Zhao H. Hua Y. Keep R. F. Xi G. (2015). White matter injury after subarachnoid hemorrhage: Role of blood-brain barrier disruption and matrix metalloproteinase-9. Stroke 46 (10), 2909–2915. 10.1161/strokeaha.115.010351 26374478 PMC4589516

[B10] Friedrich V. Flores R. Muller A. Bi W. Peerschke E. I. Sehba F. A. (2011). Reduction of neutrophil activity decreases early microvascular injury after subarachnoid haemorrhage. J. Neuroinflammation 8, 103. 10.1186/1742-2094-8-103 21854561 PMC3170601

[B11] Frontera J. A. Aledort L. Gordon E. Egorova N. Moyle H. Patel A. (2012). Early platelet activation, inflammation and acute brain injury after a subarachnoid hemorrhage: A pilot study. J. Thromb. Haemost. 10 (4), 711–713. 10.1111/j.1538-7836.2012.04651.x 22309145

[B12] Fuchs T. A. Brill A. Duerschmied D. Schatzberg D. Monestier M. Myers D. D. (2010). Extracellular DNA traps promote thrombosis. Proc. Natl. Acad. Sci. U. S. A. 107 (36), 15880–15885. 10.1073/pnas.1005743107 20798043 PMC2936604

[B13] Fujimoto M. Shiba M. Kawakita F. Liu L. Shimojo N. Imanaka-Yoshida K. (2016). Deficiency of tenascin-C and attenuation of blood-brain barrier disruption following experimental subarachnoid hemorrhage in mice. J. Neurosurg. 124 (6), 1693–1702. 10.3171/2015.4.Jns15484 26473781

[B14] Fumoto T. Naraoka M. Katagai T. Li Y. Shimamura N. Ohkuma H. (2019). The role of oxidative stress in microvascular disturbances after experimental subarachnoid hemorrhage. Transl. Stroke Res. 10 (6), 684–694. 10.1007/s12975-018-0685-0 30628008

[B15] Gao Y. Zhuang Z. Lu Y. Tao T. Zhou Y. Liu G. (2019). Curcumin mitigates neuro-inflammation by modulating microglia polarization through inhibiting TLR4 Axis signaling pathway following experimental subarachnoid hemorrhage. Front. Neurosci. 13, 1223. 10.3389/fnins.2019.01223 31803007 PMC6872970

[B16] Gris T. Laplante P. Thebault P. Cayrol R. Najjar A. Joannette-Pilon B. (2019). Innate immunity activation in the early brain injury period following subarachnoid hemorrhage. J. Neuroinflammation 16 (1), 253. 10.1186/s12974-019-1629-7 31801576 PMC6894125

[B17] Guo Z. Hu Q. Xu L. Guo Z. N. Ou Y. He Y. (2016). Lipoxin A4 reduces inflammation through formyl peptide receptor 2/p38 MAPK signaling pathway in subarachnoid hemorrhage rats. Stroke 47 (2), 490–497. 10.1161/strokeaha.115.011223 26732571 PMC4729632

[B18] Hanafy K. A. (2013). The role of microglia and the TLR4 pathway in neuronal apoptosis and vasospasm after subarachnoid hemorrhage. J. Neuroinflammation 10, 83. 10.1186/1742-2094-10-83 23849248 PMC3750560

[B19] Ishikawa M. Kusaka G. Yamaguchi N. Sekizuka E. Nakadate H. Minamitani H. (2009). Platelet and leukocyte adhesion in the microvasculature at the cerebral surface immediately after subarachnoid hemorrhage. Neurosurgery 64 (3), 546–553. 10.1227/01.Neu.0000337579.05110.F4 19240618

[B20] Jaja B. N. R. Saposnik G. Lingsma H. F. Macdonald E. Thorpe K. E. Mamdani M. (2018). Development and validation of outcome prediction models for aneurysmal subarachnoid haemorrhage: The SAHIT multinational cohort study. Bmj 360, j5745. 10.1136/bmj.j5745 29348138

[B21] Kang L. Yu H. Yang X. Zhu Y. Bai X. Wang R. (2020). Neutrophil extracellular traps released by neutrophils impair revascularization and vascular remodeling after stroke. Nat. Commun. 11 (1), 2488. 10.1038/s41467-020-16191-y 32427863 PMC7237502

[B22] Karimy J. K. Reeves B. C. Kahle K. T. (2020). Targeting TLR4-dependent inflammation in post-hemorrhagic brain injury. Expert Opin. Ther. Targets 24 (6), 525–533. 10.1080/14728222.2020.1752182 32249624 PMC8104018

[B23] Kawakita F. Fujimoto M. Liu L. Nakano F. Nakatsuka Y. Suzuki H. (2017). Effects of toll-like receptor 4 antagonists against cerebral vasospasm after experimental subarachnoid hemorrhage in mice. Mol. Neurobiol. 54 (8), 6624–6633. 10.1007/s12035-016-0178-7 27738873

[B24] Kusaka G. Ishikawa M. Nanda A. Granger D. N. Zhang J. H. (2004). Signaling pathways for early brain injury after subarachnoid hemorrhage. J. Cereb. Blood Flow. Metab. 24 (8), 916–925. 10.1097/01.Wcb.0000125886.48838.7e 15362722

[B25] Kwon M. S. Woo S. K. Kurland D. B. Yoon S. H. Palmer A. F. Banerjee U. (2015). Methemoglobin is an endogenous toll-like receptor 4 ligand-relevance to subarachnoid hemorrhage. Int. J. Mol. Sci. 16 (3), 5028–5046. 10.3390/ijms16035028 25751721 PMC4394463

[B26] Lamb J. Crawford E. D. Peck D. Modell J. W. Blat I. C. Wrobel M. J. (2006). The connectivity map: Using gene-expression signatures to connect small molecules, genes, and disease. Science 313 (5795), 1929–1935. 10.1126/science.1132939 17008526

[B27] Langfelder P. Horvath S. (2008). Wgcna: an R package for weighted correlation network analysis. BMC Bioinforma. 9, 559. 10.1186/1471-2105-9-559 PMC2631488 19114008

[B28] Lenski M. Huge V. Briegel J. Tonn J. C. Schichor C. Thon N. (2017). Interleukin 6 in the cerebrospinal fluid as a biomarker for onset of vasospasm and ventriculitis after severe subarachnoid hemorrhage. World Neurosurg. 99, 132–139. 10.1016/j.wneu.2016.11.131 27931942

[B29] Li J. Zheng S. Chen B. Butte A. J. Swamidass S. J. Lu Z. (2016). A survey of current trends in computational drug repositioning. Brief. Bioinform 17 (1), 2–12. 10.1093/bib/bbv020 25832646 PMC4719067

[B30] Li Y. Wu P. Bihl J. C. Shi H. (2020). Underlying mechanisms and potential therapeutic molecular targets in blood-brain barrier disruption after subarachnoid hemorrhage. Curr. Neuropharmacol. 18 (12), 1168–1179. 10.2174/1570159x18666200106154203 31903882 PMC7770641

[B31] Liberzon A. Subramanian A. Pinchback R. Thorvaldsdóttir H. Tamayo P. Mesirov J. P. (2011). Molecular signatures database (MSigDB) 3.0. Bioinformatics 27 (12), 1739–1740. 10.1093/bioinformatics/btr260 21546393 PMC3106198

[B32] Liu L. Fujimoto M. Nakano F. Nishikawa H. Okada T. Kawakita F. (2018). Deficiency of tenascin-C alleviates neuronal apoptosis and neuroinflammation after experimental subarachnoid hemorrhage in mice. Mol. Neurobiol. 55 (11), 8346–8354. 10.1007/s12035-018-1006-z 29546590

[B33] Lovelock C. E. Rinkel G. J. Rothwell P. M. (2010). Time trends in outcome of subarachnoid hemorrhage: Population-based study and systematic review. Neurology 74 (19), 1494–1501. 10.1212/WNL.0b013e3181dd42b3 20375310 PMC2875923

[B34] Lu Y. Zhang X. S. Zhang Z. H. Zhou X. M. Gao Y. Y. Liu G. J. (2018). Peroxiredoxin 2 activates microglia by interacting with Toll-like receptor 4 after subarachnoid hemorrhage. J. Neuroinflammation 15 (1), 87. 10.1186/s12974-018-1118-4 29554978 PMC5859544

[B35] Lucke-Wold B. P. Logsdon A. F. Manoranjan B. Turner R. C. McConnell E. Vates G. E. (2016). Aneurysmal subarachnoid hemorrhage and neuroinflammation: A comprehensive review. Int. J. Mol. Sci. 17 (4), 497. 10.3390/ijms17040497 27049383 PMC4848953

[B36] Macdonald R. L. Schweizer T. A. (2017). Spontaneous subarachnoid haemorrhage. Lancet 389 (10069), 655–666. 10.1016/s0140-6736(16)30668-7 27637674

[B37] Maddahi A. Povlsen G. K. Edvinsson L. (2012). Regulation of enhanced cerebrovascular expression of proinflammatory mediators in experimental subarachnoid hemorrhage via the mitogen-activated protein kinase kinase/extracellular signal-regulated kinase pathway. J. Neuroinflammation 9, 274. 10.1186/1742-2094-9-274 23259581 PMC3573995

[B38] Maugeri N. Campana L. Gavina M. Covino C. De Metrio M. Panciroli C. (2014). Activated platelets present high mobility group box 1 to neutrophils, inducing autophagy and promoting the extrusion of neutrophil extracellular traps. J. Thromb. Haemost. 12 (12), 2074–2088. 10.1111/jth.12710 25163512

[B39] Mauler M. Herr N. Schoenichen C. Witsch T. Marchini T. Härdtner C. (2019). Platelet serotonin aggravates myocardial ischemia/reperfusion injury via neutrophil degranulation. Circulation 139 (7), 918–931. 10.1161/circulationaha.118.033942 30586717 PMC6370531

[B40] McBride D. W. Blackburn S. L. Peeyush K. T. Matsumura K. Zhang J. H. (2017). The role of thromboinflammation in delayed cerebral ischemia after subarachnoid hemorrhage. Front. Neurol. 8, 555. 10.3389/fneur.2017.00555 29109695 PMC5660311

[B41] Naegelen I. Beaume N. Plançon S. Schenten V. Tschirhart E. J. Bréchard S. (2015). Regulation of neutrophil degranulation and cytokine secretion: A novel model approach based on linear fitting. J. Immunol. Res. 2015, 817038. 10.1155/2015/817038 26579547 PMC4633572

[B42] Nakano F. Kawakita F. Liu L. Nakatsuka Y. Nishikawa H. Okada T. (2019). Anti-vasospastic effects of epidermal growth factor receptor inhibitors after subarachnoid hemorrhage in mice. Mol. Neurobiol. 56 (7), 4730–4740. 10.1007/s12035-018-1400-6 30382533

[B43] Neifert S. N. Chapman E. K. Martini M. L. Shuman W. H. Schupper A. J. Oermann E. K. (2021). Aneurysmal subarachnoid hemorrhage: The last decade. Transl. Stroke Res. 12 (3), 428–446. 10.1007/s12975-020-00867-0 33078345

[B44] Newman A. M. Liu C. L. Green M. R. Gentles A. J. Feng W. Xu Y. (2015). Robust enumeration of cell subsets from tissue expression profiles. Nat. Methods 12 (5), 453–457. 10.1038/nmeth.3337 25822800 PMC4739640

[B45] Nishikawa H. Liu L. Nakano F. Kawakita F. Kanamaru H. Nakatsuka Y. (2018). Modified citrus pectin prevents blood-brain barrier disruption in mouse subarachnoid hemorrhage by inhibiting galectin-3. Stroke 49 (11), 2743–2751. 10.1161/strokeaha.118.021757 30355205

[B46] Okada T. Kawakita F. Nishikawa H. Nakano F. Liu L. Suzuki H. (2019). Selective toll-like receptor 4 antagonists prevent acute blood-brain barrier disruption after subarachnoid hemorrhage in mice. Mol. Neurobiol. 56 (2), 976–985. 10.1007/s12035-018-1145-2 29855971

[B47] Okada T. Suzuki H. Travis Z. D. Altay O. Tang J. Zhang J. H. (2021). SPARC aggravates blood-brain barrier disruption via integrin αvβ3/MAPKs/MMP-9 signaling pathway after subarachnoid hemorrhage. Oxid. Med. Cell Longev. 2021, 9739977. 10.1155/2021/9739977 34804372 PMC8601826

[B48] Pera J. Korostynski M. Golda S. Piechota M. Dzbek J. Krzyszkowski T. (2013). Gene expression profiling of blood in ruptured intracranial aneurysms: In search of biomarkers. J. Cereb. Blood Flow. Metab. 33 (7), 1025–1031. 10.1038/jcbfm.2013.37 23512133 PMC3705426

[B49] Plow E. F. Wang Y. Simon D. I. (2018). The search for new antithrombotic mechanisms and therapies that may spare hemostasis. Blood 131 (17), 1899–1902. 10.1182/blood-2017-10-784074 29467183 PMC5921961

[B50] Provencio J. J. Altay T. Smithason S. Moore S. K. Ransohoff R. M. (2011). Depletion of Ly6G/C(+) cells ameliorates delayed cerebral vasospasm in subarachnoid hemorrhage. J. Neuroimmunol. 232 (1-2), 94–100. 10.1016/j.jneuroim.2010.10.016 21059474 PMC3053416

[B51] Provencio J. J. Swank V. Lu H. Brunet S. Baltan S. Khapre R. V. (2016). Neutrophil depletion after subarachnoid hemorrhage improves memory via NMDA receptors. Brain Behav. Immun. 54, 233–242. 10.1016/j.bbi.2016.02.007 26872422 PMC4828315

[B52] Ridwan S. Grote A. Simon M. (2021). Interleukin 6 in cerebrospinal fluid is a biomarker for delayed cerebral ischemia (DCI) related infarctions after aneurysmal subarachnoid hemorrhage. Sci. Rep. 11 (1), 12. 10.1038/s41598-020-79586-3 33420113 PMC7794326

[B53] Ritchie M. E. Phipson B. Wu D. Hu Y. Law C. W. Shi W. (2015). Limma powers differential expression analyses for RNA-sequencing and microarray studies. Nucleic Acids Res. 43 (7), e47. 10.1093/nar/gkv007 25605792 PMC4402510

[B54] Rubio-Araiz A. Porcu F. Pérez-Hernández M. García-Gutiérrez M. S. Aracil-Fernández M. A. Gutierrez-López M. D. (2017). Disruption of blood-brain barrier integrity in postmortem alcoholic brain: Preclinical evidence of TLR4 involvement from a binge-like drinking model. Addict. Biol. 22 (4), 1103–1116. 10.1111/adb.12376 26949123

[B55] Sasaki T. Kasuya H. Onda H. Sasahara A. Goto S. Hori T. (2004). Role of p38 mitogen-activated protein kinase on cerebral vasospasm after subarachnoid hemorrhage. Stroke 35 (6), 1466–1470. 10.1161/01.Str.0000127425.47266.20 15118180

[B56] Schneider U. C. Schiffler J. Hakiy N. Horn P. Vajkoczy P. (2012). Functional analysis of Pro-inflammatory properties within the cerebrospinal fluid after subarachnoid hemorrhage *in vivo* and *in vitro* . J. Neuroinflammation 9, 28. 10.1186/1742-2094-9-28 22316109 PMC3305442

[B57] Schneider U. C. Xu R. Vajkoczy P. (2018). Inflammatory events following subarachnoid hemorrhage (SAH). Curr. Neuropharmacol. 16 (9), 1385–1395. 10.2174/1570159x16666180412110919 29651951 PMC6251050

[B58] Shannon P. Markiel A. Ozier O. Baliga N. S. Wang J. T. Ramage D. (2003). Cytoscape: A software environment for integrated models of biomolecular interaction networks. Genome Res. 13 (11), 2498–2504. 10.1101/gr.1239303 14597658 PMC403769

[B59] Sørensen O. E. Borregaard N. (2016). Neutrophil extracellular traps - the dark side of neutrophils. J. Clin. Invest 126 (5), 1612–1620. 10.1172/jci84538 27135878 PMC4855925

[B60] Suzuki H. Ayer R. Sugawara T. Chen W. Sozen T. Hasegawa Y. (2010a). Protective effects of recombinant osteopontin on early brain injury after subarachnoid hemorrhage in rats. Crit. Care Med. 38 (2), 612–618. 10.1097/CCM.0b013e3181c027ae 19851092 PMC2808465

[B61] Suzuki H. Hasegawa Y. Chen W. Kanamaru K. Zhang J. H. (2010b). Recombinant osteopontin in cerebral vasospasm after subarachnoid hemorrhage. Ann. Neurol. 68 (5), 650–660. 10.1002/ana.22102 21031580 PMC2967465

[B62] Suzuki H. Hasegawa Y. Kanamaru K. Zhang J. H. (2010c). Mechanisms of osteopontin-induced stabilization of blood-brain barrier disruption after subarachnoid hemorrhage in rats. Stroke 41 (8), 1783–1790. 10.1161/strokeaha.110.586537 20616319 PMC2923856

[B63] Swystun L. L. Liaw P. C. (2016). The role of leukocytes in thrombosis. Blood 128 (6), 753–762. 10.1182/blood-2016-05-718114 27354721

[B64] Szklarczyk D. Franceschini A. Wyder S. Forslund K. Heller D. Huerta-Cepas J. (2015). STRING v10: Protein-protein interaction networks, integrated over the tree of life. Nucleic Acids Res. 43 (0), D447–D452. 10.1093/nar/gku1003 25352553 PMC4383874

[B65] Takeuchi O. Akira S. (2010). Pattern recognition receptors and inflammation. Cell 140 (6), 805–820. 10.1016/j.cell.2010.01.022 20303872

[B66] Tatsiy O. de Carvalho Oliveira V. Mosha H. T. McDonald P. P. (2021). Early and late processes driving NET formation, and the autocrine/paracrine role of endogenous RAGE ligands. Front. Immunol. 12, 675315. 10.3389/fimmu.2021.675315 34616390 PMC8488397

[B67] Thanabalasuriar A. Scott B. N. V. Peiseler M. Willson M. E. Zeng Z. Warrener P. (2019). Neutrophil extracellular traps confine *Pseudomonas aeruginosa* ocular biofilms and restrict brain invasion. Cell Host Microbe 25 (4), 526–536. 10.1016/j.chom.2019.02.007 30930127 PMC7364305

[B68] Trinks U. Buchdunger E. Furet P. Kump W. Mett H. Meyer T. (1994). Dianilinophthalimides: Potent and selective, ATP-competitive inhibitors of the EGF-receptor protein tyrosine kinase. J. Med. Chem. 37 (7), 1015–1027. 10.1021/jm00033a019 8151612

[B69] Tso M. K. Macdonald R. L. (2014). Subarachnoid hemorrhage: A review of experimental studies on the microcirculation and the neurovascular unit. Transl. Stroke Res. 5 (2), 174–189. 10.1007/s12975-014-0323-4 24510780

[B70] Vaibhav K. Braun M. Alverson K. Khodadadi H. Kutiyanawalla A. Ward A. (2020). Neutrophil extracellular traps exacerbate neurological deficits after traumatic brain injury. Sci. Adv. 6 (22), eaax8847. 10.1126/sciadv.aax8847 32523980 PMC7259928

[B71] Vallés J. Lago A. Santos M. T. Latorre A. M. Tembl J. I. Salom J. B. (2017). Neutrophil extracellular traps are increased in patients with acute ischemic stroke: Prognostic significance. Thromb. Haemost. 117 (10), 1919–1929. 10.1160/th17-02-0130 28837206

[B72] Vikman P. Ansar S. Edvinsson L. (2007). Transcriptional regulation of inflammatory and extracellular matrix-regulating genes in cerebral arteries following experimental subarachnoid hemorrhage in rats. Laboratory investigation. J. Neurosurg. 107 (5), 1015–1022. 10.3171/jns-07/11/1015 17977275

[B73] Wang R. Zhu Y. Liu Z. Chang L. Bai X. Kang L. (2021). Neutrophil extracellular traps promote tPA-induced brain hemorrhage via cGAS in mice with stroke. Blood 138 (1), 91–103. 10.1182/blood.2020008913 33881503 PMC8288643

[B74] Wang Y. Gao H. Shi C. Erhardt P. W. Pavlovsky A. D A. S. (2017). Leukocyte integrin Mac-1 regulates thrombosis via interaction with platelet GPIbα. Nat. Commun. 8, 15559. 10.1038/ncomms15559 28555620 PMC5477519

[B75] Weisenburger-Lile D. Dong Y. Yger M. Weisenburger G. Polara G. F. Chaigneau T. (2019). Harmful neutrophil subsets in patients with ischemic stroke: Association with disease severity. Neurol. Neuroimmunol. Neuroinflamm 6 (4), e571. 10.1212/nxi.0000000000000571 31355307 PMC6624098

[B76] Wink M. (2007). Molecular modes of action of cytotoxic alkaloids: From DNA intercalation, spindle poisoning, topoisomerase inhibition to apoptosis and multiple drug resistance. Alkaloids Chem. Biol. 64, 1–47. 10.1016/s1099-4831(07)64001-2 18085328

[B77] Wu F. Liu Z. Li G. Zhou L. Huang K. Wu Z. (2021). Inflammation and oxidative stress: Potential targets for improving prognosis after subarachnoid hemorrhage. Front. Cell Neurosci. 15, 739506. 10.3389/fncel.2021.739506 34630043 PMC8497759

[B78] Yang C. Zhang H. Chen M. Wang S. Qian R. Zhang L. (2022). A survey of optimal strategy for signature-based drug repositioning and an application to liver cancer. Elife 11. 10.7554/eLife.71880 PMC8893721 35191375

[B79] Yu F. Saand A. Xing C. Lee J. W. Hsu L. Palmer O. P. (2021). CSF lipocalin-2 increases early in subarachnoid hemorrhage are associated with neuroinflammation and unfavorable outcome. J. Cereb. Blood Flow. Metab. 41 (10), 2524–2533. 10.1177/0271678x211012110 33951946 PMC8504948

[B80] Yu G. Wang L. G. Han Y. He Q. Y. (2012). clusterProfiler: an R package for comparing biological themes among gene clusters. Omics 16 (5), 284–287. 10.1089/omi.2011.0118 22455463 PMC3339379

[B81] Zeng H. Fu X. Cai J. Sun C. Yu M. Peng Y. (2022). Neutrophil extracellular traps may be a potential target for treating early brain injury in subarachnoid hemorrhage. Transl. Stroke Res. 13 (1), 112–131. 10.1007/s12975-021-00909-1 33852132

[B82] Zhang X. Lu Y. Wu Q. Dai H. Li W. Lv S. (2019). Astaxanthin mitigates subarachnoid hemorrhage injury primarily by increasing sirtuin 1 and inhibiting the Toll-like receptor 4 signaling pathway. Faseb J. 33 (1), 722–737. 10.1096/fj.201800642RR 30048156

[B83] Zhang Y. Li L. Jia L. Li T. Di Y. Wang P. (2021). Neutrophil counts as promising marker for predicting in-hospital mortality in aneurysmal subarachnoid hemorrhage. Stroke 52 (10), 3266–3275. 10.1161/strokeaha.120.034024 34167330

