# Supplementary material for: Specific signature biomarkers highlight the potential mechanisms of circulating neutrophils in aneurysmal subarachnoid hemorrhage
Source: Front Pharmacol. 2022 Nov 10;13:1022564. doi: 10.3389/fphar.2022.1022564 (PMC9685413; doi:10.3389/fphar.2022.1022564)
Supplement: Supplementary file 1 [file Image1.pdf]

Supplement

Figure S1

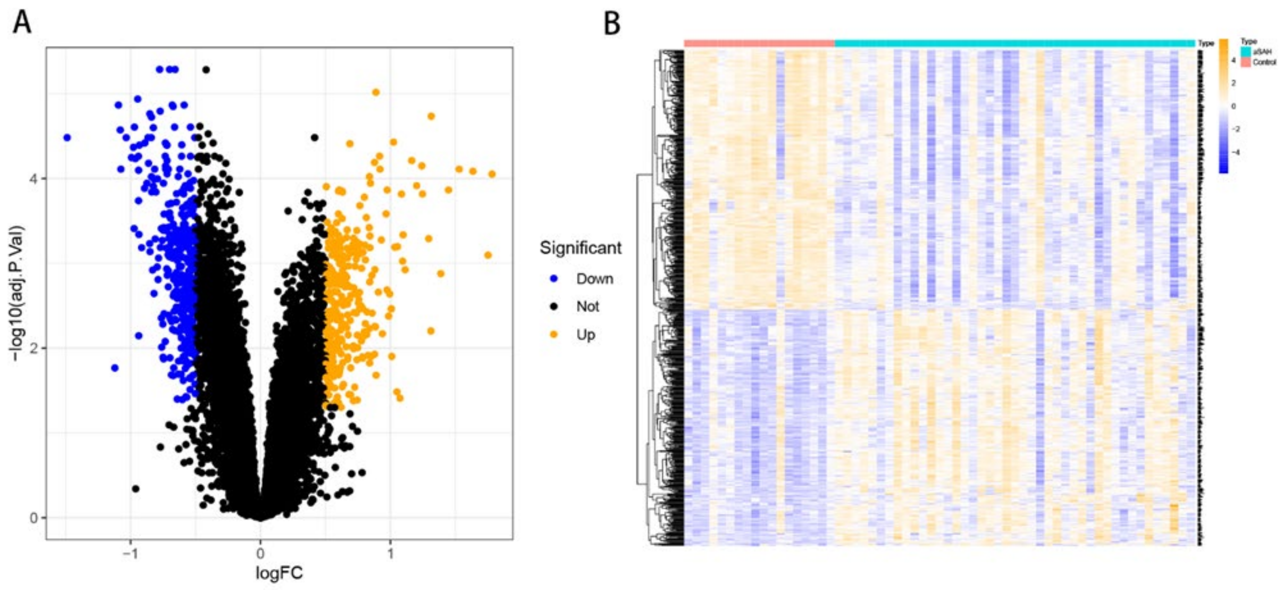

Legend: Identification of DEGs visualized the volcano plot (A) and heatmap (B). Orange indicates upregulation, while blue indicates downregulation.
